# OPN Induces FoxM1 Expression and Localization through ERK 1/2, AKT, and p38 Signaling Pathway in HEC-1A Cells

**DOI:** 10.3390/ijms151223345

**Published:** 2014-12-16

**Authors:** Yunpeng Xie, Yinghua Li, Ying Kong

**Affiliations:** Department of Biochemistry and Molecular, Dalian Medical University, Dalian 116044, China; E-Mails: yunpeng_xie1986@163.com (Y.X.); yinghua_li1989@163.com (Y.L.)

**Keywords:** FoxM1, OPN, proliferation

## Abstract

Mammalian embryo implantation is an extremely complex process and requires endometrial receptivity. In order to establish this receptivity, sequential proliferation and differentiation during the menstrual cycle is necessary. Forkhead box M1 (FoxM1) is described as a major oncogenic transcription factor in tumor initiation, promotion and progression. According to these functions, we believe that FoxM1 should also play an essential role in embryo implantation. Osteopontin (OPN), an adhesion molecule, has been studied extensively in reproduction. In this study, we observed the expression and distribution of FoxM1 during the proliferative-phase and secretory-phase human endometrium and the pre-implantation mouse uterus firstly. Then we observed the relationship between OPN and FoxM1. Our results showed that FoxM1 was mainly distributed in glandular epithelium. OPN increased the expression of FoxM1 in the human uterine epithelial cell line HEC-1A cells in a time- and concentration-dependent manner. OPN regulates FoxM1 to influence HEC-1A cell proliferation through extracellular regulated protein kinases (ERK 1/2), protein kinase B (PKB, AKT), and the p38 mitogen activated protein kinases (p38MAPK, p38) signaling pathway. Inhibition of ERK 1/2, AKT and p38 suppressed OPN-induced FoxM1 expression and location. Our data indicate that FoxM1 might be regulated by OPN to influence endometrial proliferation to establish endometrial receptivity.

## 1. Introduction

Embryo implantation is an extremely complex process, including apposition, adhesion, penetration and trophoblast invasion. Implantation failure is one of the major reasons for infertility and remains an obstacle to the progress of assisted reproductive techniques [1]. Embryo implantation only occur during the implantation window, when the blastocyst is accepted by the maternal endometrium through mediation by adhesion molecules, immune cells, cytokines, growth factors, chemokines and so on [2].

Osteopontin (OPN) is an extracellular matrix (ECM) molecule and is involved in many physiologic and pathologic processes, including cell adhesion [3], angiogenesis [4] and tumor metastasis [5]. In reproduction, the role of OPN has been studied extensively [6,7,8]. OPN, as one of the adhesion molecules, play a role in the various stages of blastocyst implantation [9]. In women, OPN is expressed in glandular epithelial cells and in increasing concentrations in uterine secretions during the mid to late secretory phase [10].

Forkhead box M1 (FoxM1), as a member of Forkhead family of transcription factors, shares homology in Winged Helix/Forkhead box DNA-binding domain [11]. It has been recognized that FoxM1 is involved in cell proliferation and apoptosis which regulates the developmental function of many organs in the body [12]. Several lines of evidence demonstrate that overexpression of FoxM1 occurs in a wide variety of human tumors frequently, including medulloblastoma [13], colorectal cancer [14], hepatocellular carcinoma [15], breast cancer [16], non-small cell lung cancer [17] and so on. Embryo implantation and cancer follow a similar progression and molecular mechanisms, such as epigenetic processes and dynamic regulation of cell migration and invasion [18]. So, given the similarity between the progress of tumor progression and embryo implantation, we presume that FoxM1 may be an indispensable factor in embryo implantation. Our previous study had proved that FoxM1 could be regulated by estrogen and progesterone and influenced embryo implantation [19]. We used human uterine epithelial cell line HEC-1A as *in vitro* models. HEC-1A cells are used as a model of non-receptive endometrium.

In this study, we demonstrate that OPN upregulated the expression of FoxM1 to influence the proliferation of HEC-1A cells.

## 2. Results

### 2.1. Expression of Forkhead Box M1 (FoxM1) in Human Endometrial Tissues

**Figure 1:**
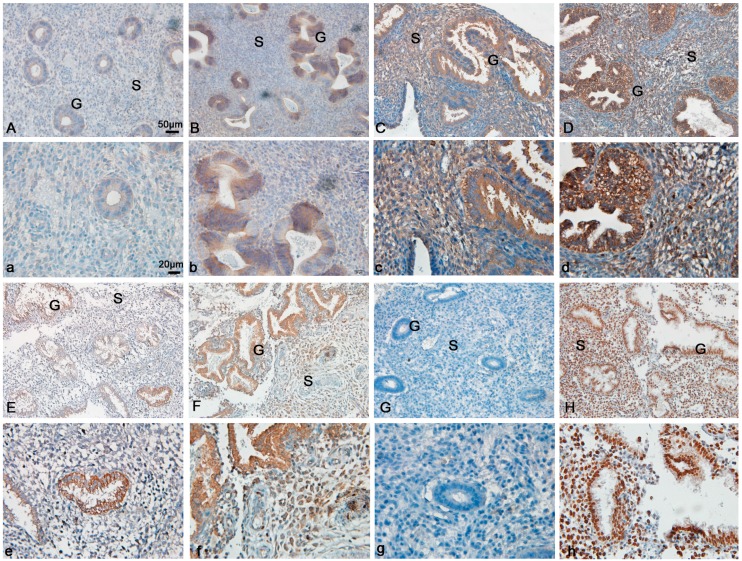
Expression of Forkhead box M1 (FoxM1) in the proliferative stage and the secretory stage of human endometrial tissues, stromal cells (S), glandular epithelium (G), FoxM1 expression in early proliferative phase (**A**,**a**); in the mid-proliferative stage (**B**,**b**); in the late-proliferative stage (**C**,**c**); in the early secretory stage (**D**,**d**); in the mid-secretory stage (**E**,**e**); in the late secretory (**F**,**f**); negative control (**G**,**g**); positive control (**H**,**h**). (**A**–**F**): 20×; (**a**–**f**): 40×.

Immunohistochemistry was performed to examine the distribution of FoxM1 protein in the human endometrium during the proliferative- and secretory-phases. As shown in Figure 1 and Table 1, in the early proliferative phase, FoxM1 was minimally expressed (Figure 1A,a). In the mid-proliferative stage, FoxM1 was highly expressed in the glandular epithelia and stromal cells (Figure 1B,b). In the late-proliferative stage and early secretory stage, FoxM1 was also expressed strongly both in glandular epithelia and stromal cells (Figure 1C,c,D,d). In the mid-secretory stage, the expression of FoxM1 was weak (Figure 1E,e), while it recovered a bit in the late secretory stage (Figure 1F,f).

**Table 1:** Expression of FoxM1 protein image analysis in human endometrial epithelium of different phases.

MC	EP	MP	LP	ES	MS	LS
*H*-score	0.12 ± 0.02	0.89 ± 0.09	1.8 ± 0.16	2.5 ± 0.22	1.4 ± 0.16	1.8 ± 0.20

MC, menstrual cycle; EP, early proliferative phase; MP, mid-proliferative phase; LP, late-proliferative phase; ES, early secretory phase; MS, mid-secretory phase; LE, late-secretory phase. Eight specimens each from different phases. Results were expressed as the mean + SEM of *H*-score.

### 2.2. Expression of FoxM1 in Mouse Uterus during Early Pregnancy

**Figure 2:**
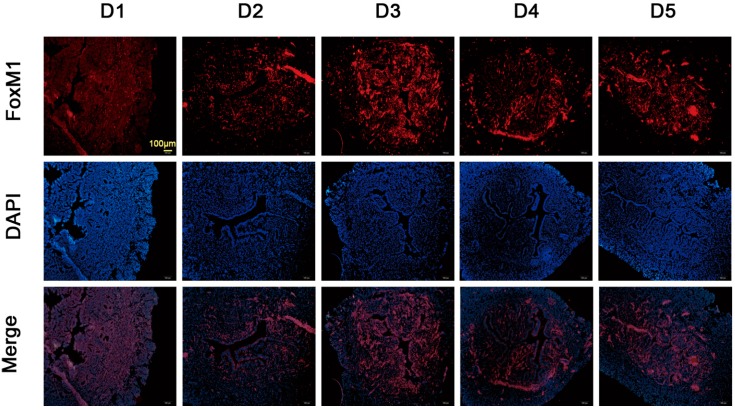
Expression of FoxM1 in mouse uterus during early pregnancy. Females were checked for the presence of a vaginal plug in the next morning, which was defined as Day 1 (D1) if the vaginal plug came out. The mice were killed on D1–D5, as indicated. Bar = 100 μm.

As shown in Figure 2, FoxM1 was mainly located in the glandular epithelium, luminal epithelium and stromal cells on Day 3. FoxM1 was located in stromal cells on Day 4 and was located in glandular epithelium and luminal epithelium on Day 5.

### 2.3. Osteopontin (OPN) Induces FoxM1 Expression in HEC-1A Cells

**Figure 3:**
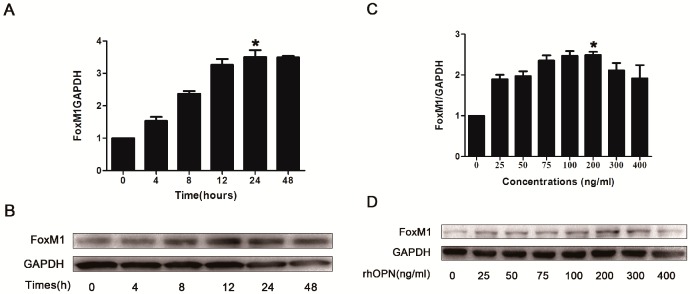
Recombinant human OPN (rhOPN) induces FoxM1 expression (**A**,**B**). HEC-1A cells were treated with various concentrations of rhOPN for 24 h; (**C**,**D**) HEC-1A cells were treated for various periods with rhOPN at a concentration of 200 ng/mL. * *p* < 0.05.

To determine whether OPN induces FoxM1 expression, HEC-1A cells were treated without or with recombinant human OPN (rhOPN) (200 ng/mL) for 4, 8, 12, 24, 48 h and the expression of FoxM1 was detected by Real-Time PCR and western blot. The results indicated that rhOPN induced FoxM1 expression in a time-dependent manner in HEC-1A cells, and the protein expression of FoxM1 reached the peak at the point of 24 h (Figure 3A,B). We also studied the dose-dependent response of rhOPN (24 h) for 0, 25, 50, 75, 100, 200, 300 and 400 ng/mL. It showed that the protein expression of FoxM1 increased in a dose-dependent manner and that 200 ng/mL rhOPN induced the highest level of FoxM1 expression compared with other doses (Figure 3C,D).

### 2.4. OPN Induces FoxM1 Protein Expression through Extracellular Regulated Protein Kinases (ERK 1/2), Protein Kinase B (PKB, AKT), and the p38 Mitogen Activated Protein Kinases (p38MAPK, p38) Signaling Pathway

**Figure 4:**
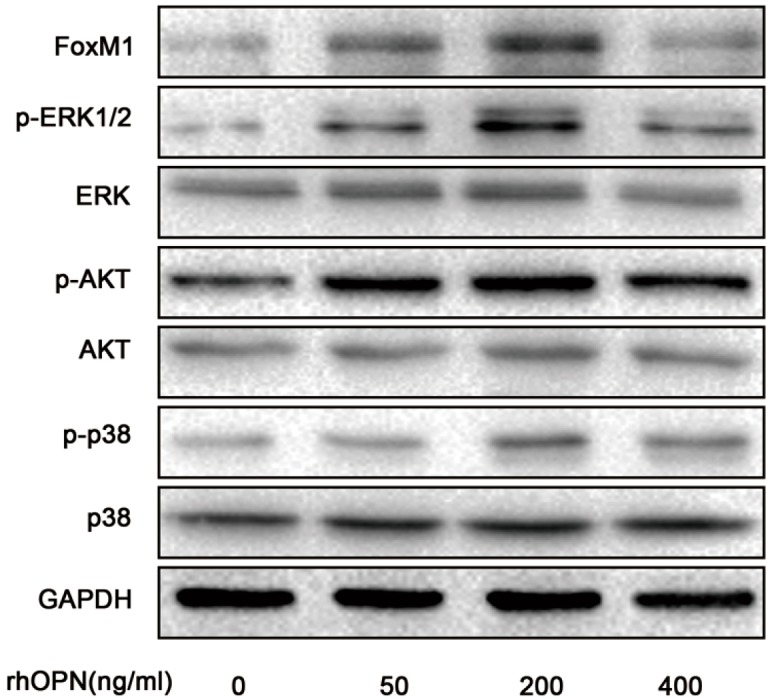
rhOPN induces FoxM1 expression through activating extracellular regulated protein kinases (ERK 1/2), protein kinase B (PKB, AKT), and p38 mitogen activated protein kinases (p38MAPK, p38). HEC-1A cells were treated with different concentrations of progesterone (0, 50, 200, 400 ng/mL).

To examine how rhOPN upregulated the expression of FoxM1, we measured the activation of extracellular regulated protein kinases (ERK 1/2), protein kinase B (PKB, AKT), and the p38 mitogen activated protein kinases (p38MAPK, p38) signaling pathway by rhOPN treatment (0, 50, 200, 400 ng/mL). The results showed that FoxM1, phosphor-ERK 1/2, phosphor-AKT (ser473) and phosphor-p38 increased after rhOPN treatment and reached a peak at 200 ng/mL. (Figure 4).

**Figure 5:**
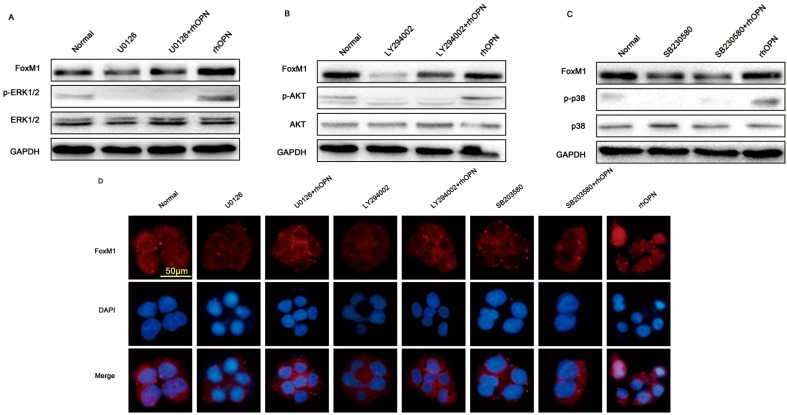
OPN-activated AKT, ERK1/2 and p38 signaling pathways influence FoxM1 expression and nuclear localization. (**A**) Treated with U0126 (a specific inhibitor of mitogen activated protein kinase kinase (MEK)/ERK; 10^−5^ M); (**B**) Treated with LY294002 (a specific inhibitor of phosphatidylinositol 3-kinase (PI3K)/AKT; 10^−6^ M); (**C**) Treated with SB203580 (a specific inhibitor of p38; 10^−6^ M); (**D**) Inhibitors suppress FoxM1 nuclear localization. Magnification: 40×; Bar = 50 μm.

To further study the mechanism of rhOPN-induced FoxM1 expression, additions of U0126 (a specific inhibitor of mitogen activated protein kinase kinase (MEK)/extracellular signal regulated kinase (ERK); 10^−5^ M), SB203580 (a specific inhibitor of p38; 10^−6^ M) and LY294002 (a specific inhibitor of phosphatidylinositol 3-kinase (PI3K)/AKT; 10^−6^ M) were separately used to pretreat cells. The results showed that treatment with U0126, SB203580 and LY294002 inhibited the phosphorylation of ERK1/2, p38 and AKT in the OPN-added cells and the expression of FoxM1 (Figure 5A–C).

FoxM1, as a transcription factor, should be in the nucleus to activate the downstream target genes and proliferation-related cell signaling pathways. We used indirect immunofluorescence staining to investigate whether the above three inhibitors influence FoxM1 to transport to nucleus. As result in Figure 5D, after treating cells with U0126, SB203580 and LY2094002, FoxM1 was not detected in the cell nucleus, even when treated with rhOPN.

### 2.5. FoxM1 Regulates Cell Proliferation in HEC-1A Cells

**Figure 6:**
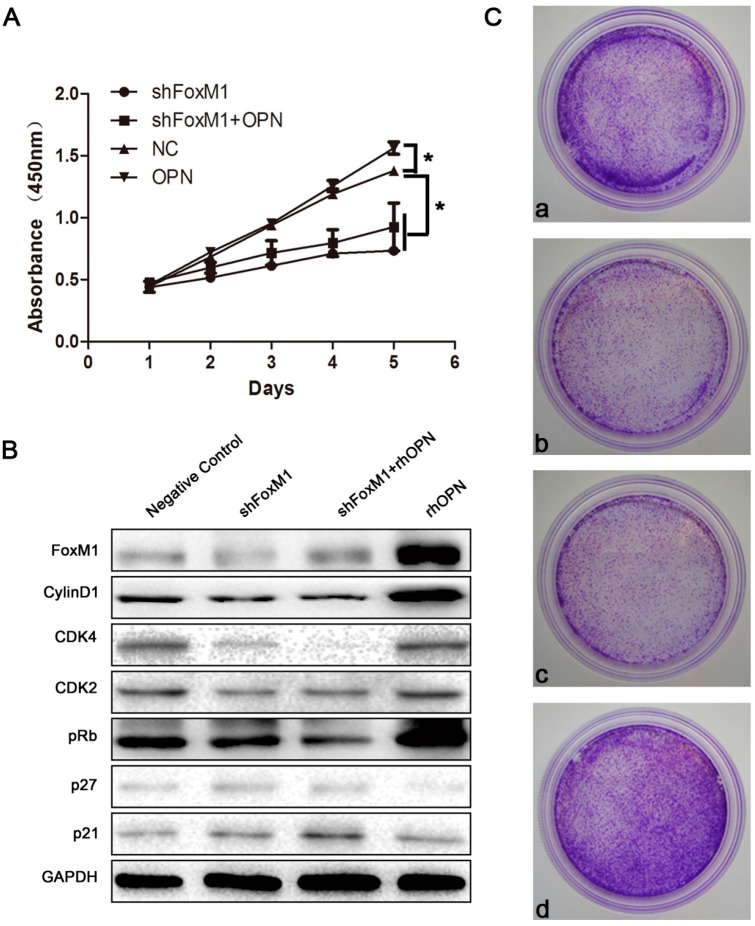
FoxM1 influences the proliferation of HEC-1A. (**A**) Cells were seeded in a 96-well plate and grown for 1–5 days. Cell proliferation was determined daily by Cell Counting Kit-8 (CCK-8) assay. * *p* < 0.05; (**B**) Effect of FoxM1 induced expression of cell cyclins and cell cycle related regulatory proteins; (**C**) Colony Formation Assay. a, NC; b, shFoxM1; c, shFoxM1 + OPN; d, OPN.

To confirm that OPN regulation of HEC-1A profileration occurs through its influence on the expression of FoxM1, we used Cell Counting Kit-8 (CCK-8) to detect the proliferating ability of HEC-1A. FoxM1 was knocked down by transient transfection of FoxM1 shRNA. As shown in Figure 6A, the groups of knockdown FoxM1 dramatically decreased cell proliferation with or without OPN treatement for 3 days in culture (* *p* < 0.05), compared with control, and proliferation was higher for the OPN-treatment group than the control group (* *p* < 0.05).

To determine the impact of FoxM1 on cell cycle progression specifically, the protein levels of cyclinD1, Cyclin-dependent kinase 4 (CDK4), Cyclin-dependent kinase 2 (CDK2), retinoblastoma protein (pRb), p27 and p21 were measured. As shown in Figure 6B, the expression of cyclinD1, CDK4, CDK2 and pRb were decreased, and the expression of p27 and p21 were increased in the groups of knockdown FoxM1 with or without OPN, compared to control.

Using a colony formation assay, we found again that shFoxM1 (FoxM1 shRNA was transiently transfected into the cells) dramatically decreased cell proliferation with or without OPN treatment for 3 days in culture, compared with control, and proliferation in the group with OPN-treatment was higher than in the control group (Figure 6C).

## 3. Discussion

Successful embryo implantation requires crosstalk between the embryo and the uterus [2]. The process of implantation consists of three stages: apposition, attachment, and invasion of the uterine luminal epithelium [20]. Although several molecular pathways of embryo implantation have been identified, a comprehensive understanding of the implantation process is still lacking [21].

In humans, the crosstalk between the blastocyst and the uterus can only occur during a brief period, namely the “window of implantation”. In mice, the blastocyst escapes from its zona pellucida and attaches to the uterine epithelium at D4.5 of pregnancy [20,22]. In this study, FoxM1 was expressed ever-increasing in the glandular epithelia during the proliferative phase. During the early secretory phase, FoxM1 was expressed strongly in the glandular epithelia and stromal cells. However, during the mid-secretory phase, FoxM1 was declining in expression and mainly distributed in the glandular epithelia. The expression of FoxM1 was higher in the late secretory phase than the mid-secretory phase (Figure 1). In mouse endometrial tissues, FoxM1 was highly expressed on D3 (Figure 2). Altogether, these results suggest that FoxM1 might be necessary for the establishment of endometrial receptivity. FoxM1, as a transcriptional factor, regulates cell proliferation and apoptosis in many organs [23]. Estrogens are essential steroid hormones that prepare the human uterine endometrium for embryo implantation and pregnancy [24,25], and activate transcription of target genes through binding their cognate receptors, the estrogen receptor (ER). FoxM1 is a physiological regulator of ERα expression in breast cancer cells [16,26]. It might be thought that estrogen can influence endometrium proliferation through regulating the expression of FoxM1.

OPN is a SIBLING (small integrin-binding ligand, *N*-linked glycoprotein) that was first identified in 1986 in osteoblasts. In the endometrium, it binds to integrin β3, giving rise to speculation that it may mediate trophoblast endometrial interactions. It is clear that secreted OPN is available as a ligand for the integrin αvβ3 heterodimer on the trophectoderm and uterus to induce adhesion between luminal epithelium and the trophectoderm essential for implantation and placentation [9,27].

A mature embryo having the capacity of adhesion and penetration needs not only to express matrix molecules such as collagen and laminin [28], but also to acquire penetrability through matrix metallo proteinases (MMPs) that degrade the extracellular matrix (ECM). MMPs are the mark of blastocyst trophoblast invasion [29,30]. The expression of MMP-2 and MMP-9 are significantly decreased in FoxM1 gene knockout cells, such as AsPC-1, Colo-357 and PANC-1 [31]. Vascular endothelial growth factor (VEGF) is essential for embryonic vasculogenesis and angiogenesis, as well as tumor angiogenesis [32,33]. Successful implantation needs the proper level of VEGF expression [34,35]. Over-expression of FoxM1b by gene transfer significantly promotes the growth and metastasis of gastric cancer cells in orthotopic mouse models [36]. FoxM1 contributes to glioma progression by enhancing VEGF gene transcription and tumor angiogenesis [37]. Therefore FoxM1 might up-regulate the expression of MMPs and VEGF, EMT to increase the capacity of cancer invasion and influence embryo implantation.

OPN has been reported to activate PI3K/AKT, p38 and ERK1/2 signaling to regulate proliferation, migration, invasion and angiogenesis [38,39]. In this study, we focused on the possible connection between OPN and FoxM1. We found that OPN increased the expression of FoxM1 in HEC-1A cells in a time- and concentration-dependent manner (Figure 3A–D). Treatment with inhibitors of ERK1/2, p38 and AKT inhibited the expression of FoxM1 in the rhOPN-added cells (Figure 4 and Figure 5A–C). Additionally, using an indirect immunofluorescence staining assay, we found that FoxM1 was not translocated into the nucleus (Figure 5D). Therefore the downstream signaling, cyclinD1, CDK2, CDK4, and pRb cannot be activated, and also, p21, and p27 cannot be inhibited (Figure 6B); resulting in suppressed proliferation of HEC-1A cells (Figure 6A,C).

## 4. Materials and Methods

### 4.1. Materials

Human uterine epithelial cell line HEC-1A cells were acquired from the American Type Culture Collection. Recombinant OPN protein (rhOPN) was purchased from R&D Systems (Minneapolis, MN, USA). Rabbit polyclonal anti-FoxM1 antibody was purchased from Santa Cruz Biotechnology (Dallas, TX, USA). Rabbit anti-AKT, phosphor-ERK 1/2, phosphor-AKT (Ser473) antibodies and mouse anti-p38, phosphor-p38 were purchased from Beyotime Institute of Biotechnology (Nantong, China). Rabbit anti-ERK, phospho-ERK 1/2, glyceraldehyde 3-phosphate dehydrogenase (GAPDH) antibodies and U0126 (a specific inhibitor of MEK/ERK; 10^−5^ M), LY294002 (a specific inhibitor of p38; 10^−6^ M), SB203580 (a specific inhibitor of PI3K/AKT; 10^−6^ M) were purchased from Bioworld Technology (Nanjing, China). FoxM1 shRNA was purchased from Genepharma (Shanghai, China). Lipofectamine TM Reagent was purchased from Invitrogen (Waltham, MA, USA). Cell Counting Kit-8 was was purchased from Dojindo (Minato-ku, Tokyo, Japan). Enhanced chemiluminescence (ECL) assay kit was purchased from Amersham (Pittsburgh, CP, USA). Horseradish peroxidase (HRP)-conjugated anti-rabbit secondary antibody, anti-mouse secondary antibody, biotinylated secondary antibody, streptavidin-horseradish peroxidase and diaminobenzidine (DAB)-peroxidase substrate were purchased from ZSGB-BIO (Beijing, China). Tetraethyl rhodamine isothiocyanate (TRITC)-conjugated goat anti-rabbit secondary antibody IgG and FITC-conjugated goat anti-mouse secondary antibody were purchased from Thermo (Waltham, MA, USA). RNA PCR Kit (AMV) version 3.0, SYBR Premix Ex Taq were purchased from Takara (Dalian, China).

### 4.2. Tissue Collection

The protocol for human study was approved by the Institutional Review Board of Dalian Medical University (Liaoning, China). All human specimens used in this study were collected from patients between the ages of 30 and 45 from 2011 to 2013, with their consent; endometrial blocks were obtained from 48 hysterectomy specimens (including eight specimens each from early proliferative phase, mid-proliferative phase, late proliferative phase, early secretory phase, mid-secretory phase, late secretory phase).

### 4.3. Animals

Mice of Kunming species were from Lab Animal Center in Dalian Medical University of China. All experimental procedures involves in the mouse studies were approved by the Institutional Review Board in Dalian Medical University (SCXK-2013-0003, December 2013, Liaoning, China). Adult female mice weighted 20–24 g and adult male mice weighted 40–44 g were maintained under controlled environmental conditions. The mice were housed at a temperature of 22–25 °C, humidity 60%, and light-controlled (12 h light, 12 h darkness) with *ad libitum* access to water and food. Females were placed with males (one female with one male per cage). Females were checked for the presence of a vaginal plug in the next morning, which was defined as D1 if the vaginal plug came out. The mice were killed in D1–D5 respectively.

### 4.4. Cell Culture

HEC-1A cells was grown in McCoy’s 5A supplement with 10% fetal bovine serum (FBS), 100 U/mL penicillin and 100 μg/mL streptomycin. Cells were maintained at 37 °C under 5% CO_2_ in humidified air.

### 4.5. Transient Transfection

After trypsinization, HEC-1A (1 × 10^6^) cells were seeded onto six-well plates. When cells reached 80% confluence, FoxM1 shRNA and negative control was transiently transfected into the cells using 400 ng of plasmid and 4 μL of LipofectamineTM reagent, following the manufacturer’s instructions. The shRNA was removed 6 h later and the cells were harvested after 72 h for protein. Three independent experiments were performed.

### 4.6. Immunohistochemistry

Serial sections (6 μm) were prepared from paraffin-embedded tissues. The sections were fixed at 60 °C for 3 h, deparaffinized in xylene and rehydrated in graded alcohol. The slides were microwaved (Defrost) for 25 min in citrate buffer in order to unmask antigen and were washed with phosphate-buffered saline (PBS) after cooling for one hour. Slides were incubated in 3% H_2_O_2_ for 20 min to block endogenous peroxidase activity. After washing in PBS, sections were blocked with blocking buffer supplemented with normal goat serum at 37 °C for 15 min to eliminate non-specific binding of conjugated secondary antibodies before incubation overnight at 4 °C with FoxM1 antibody (1:100), IgG as a negative control, ER antibody as a positive control. After washing with PBS, sections were incubated with biotinylated secondary antibody for 40 min at 37 °C. Sections were washed with PBS, then were incubated with streptavidin-horseradish peroxidase 40 min at 37 °C. Positive reactions were visualized with a 3,3*N*-Diaminobenzidine Tertrahydrochloride (DAB)-peroxidase substrate and counterstaining with haematoxylin for 30 s. Photomicrographs were taken using OLYMPUS TH4-200 microscopy (Tokyo, Japan). Histological and immunohistochemical evalutions were performed independently by two pathologists. Immunostaining intensity was evaluated in each endometrial compartment (glandular epithelium and stromal cells) using a semiquantitative method. Each sample was given a score in which both the intensity of the staining (none staining = 0; low staining = 1; medium staining = 2; strong staining = 3) and the percentage of stained cells stained cells were multiplied. In normal endometrium, the total score was calculated per compartment per sample, as follows:

*H*-score= ∑*Pi* (*i* + 1) (1)

where *i* is the intensity of staining of staining from 0 (none) to 3 (strong), and *Pi* is the percentage of stained cells for each given *i* (0%–100%).

### 4.7. RNA Isolation and Real-Time PCR

Total RNA was isolated using Trizol reagent according to the manufacturer’s instructions. The cDNA was synthesized using a RNA PCR Kit (AMV) version 3.0. Real-time PCR was performed with an Applied Biosystems Inc. (ABI, Beijing, China) Step One Plus Real-time PCR system according to the manufacturers’ recommendations. Real-time PCR reaction contained 10 μL 2× SYBR Premix Ex Taq, 0.8 μL primer mix, 0.4 μL 50× ROX Reference Dye II, 4 μL cDNA, and 4.8 μL deionized water to make a total volume of 20 μL. The relative amount of specific mRNA was normalized to glyceraldehyde-3-phosphate dehydrogenase (GAPDH). All PCR reactions were run in triplicate and were performed with 40 cycles. The results analysis was carried out using the 2^−ΔΔ*C*t^ method. The primers used were as follows. *FoxM1*: 5'-CGTCGGCCACTGATTCTCAAA-3' (forward), and 5'-GGCAGGGGATCTCTTAGGTTC-3' (reverse); *GAPDH*: 5'-GTGAAGGTCGGAGTCAACG-3' (forward), and 5'-TGAGGTCAATGAAGGGGTC-3' (reverse).

### 4.8. Western Blot

Cells were washed in PBS before incubation with Lysis Buffer (1% Triton X-100, 150 mM NaCl, 10 mM Tris, pH 7.4, 1 mM EDTA, 1 mM EGTA, pH 8.0, 0.2 mM Na_3_VO_4_, 0.2 mM phenylmethylsulfonyl fluoride, 0.5% Nonidet P-40) on ice for 10 min. The cell lysates were clarified by centrifugation at 9000× *g* for 15 min and the supernatants were collected. Protein concentration was determined with the Coomassie Protein Assay reagent using bovine serum albumin (BSA) as a standard. Equal amounts of protein extracts (30 μg) were separated by 12% sodium dodecyl sulphate (SDS)-polyacrylamide gel electrophoresis (PAGE) and transferred to nitrocellulose filter (NC) membranes. The membranes were blocked in 5% non-fat milk in Tris-buffered saline containing 0.1% Tween 20 (TBST) for 2 h at room temperature and probed with primary antibodies overnight at 4 °C. The membranes were washed with TBST three times. Then the membranes were incubated with horseradish peroxidase-conjugated antibody for 1 h at room temperature. After four washes with TBST, the membranes were processed using enhanced chemoluminescent (ECL) and visualized using Bio-Rad Laboratories. Western blots shown are representative of at least three independent experiments. Densitometry of each band for the target protein was quantified by densitometry analysis with Labworks 4.6 (Media Cybernetics Inc., Bethesda, MD, USA). The protein band intensity was quantified by the mean ± SEM of three experiments for each group as determined from densitometry relative to GAPDH (1:5000).

### 4.9. Indirect Immunofluorescence Staining

After washing with PBS, cells grown on coverslips were fixed in 4% paraformaldehyde following 20 min and washed in PBS three times. Nonspecific binding sites were blocked by incubation with 1% goat serum in PBS at room temperature for 1 h. Slides were incubated with the FoxM1 antibody (1:100) overnight at 4 °C. After washing three times with PBS, slides were incubated with Tetraethyl rhodamine isothiocyanate (TRITC) conjugated goat anti-rabbit antibody (1:100) for 1 h at 37 °C. After three washes, slides were incubated with 4',6-diamidino-2-phenylindole (DAPI) for 5 min at room temperature and washed three times again. Specimens were mounted in PBS containing 90% glycerol and 1.0% *P*-phenylenediamine and subsequently monitored under an Olympus BX51 immunofluorescence microscope.

### 4.10. Cell Proliferation Assay

Cell proliferation was detected by a Cell Counting Kit-8 assay. HEC-1A cells were suspended in McCoy’s 5A medium supplemented with 15% heat-inactivated fatal bovine serum and subsequently seeded in 96-well plates and incubated for 24 h. After that, plates were divided into four groups: (1) negative group; (2) Transfected with FoxM1 shRNA group; (3) Transfected with FoxM1 shRNA and added rhOPN group; (4) Added rhOPN group. Then, plates were continued incubating 0, 24, 48, 72 h. After incubation, 10 μL CCK-8 solution was added to the cultures in each well and incubated at 37 °C for another 2 h. Optical density (OD) value of absorbance at 450 nm was measured by Thermo Scientific Fluoroskan Ascent FL (Waltham, MA, USA). The results were plotted as means ± SD of three independent experiments having three determinations per sample for each experiment.

### 4.11. Colony Formation Assay

Cells were digested in 0.25% trypsin to reconstitute the single cell suspension at a density of 10^5^ cells per mL. Cell suspensions were transferred into 60 mm plates and divided into four groups for the cell proliferation assay, then incubated at 37 °C at an atmosphere of 5% CO_2_ for 10 days. The supernatants were discarded, and cells were rinsed twice in PBS and fixed in methanol for 10 min. The cells were stained with crystal violet and allowed to air dry at room temperature.

### 4.12. Statistical Analysis

Each experiment was repeated 3–6 times, with results presented as the mean ± SEM. Statistical differences between test groups were analysed by one-way analysis of variance and Student-Newman–Keuls *q* value tests; *p* < 0.05 was considered to be significant.

## 5. Conclusions

In conclusion, FoxM1 increased cell proliferation by OPN influence on p38, ERK1/2, and AKT signaling pathways in HEC-1A cells.
